# Intelligent computing through neural networks for numerical treatment of non-Newtonian wire coating analysis model

**DOI:** 10.1038/s41598-021-88499-8

**Published:** 2021-04-27

**Authors:** Jawaher Lafi Aljohani, Eman Salem Alaidarous, Muhammad Asif Zahoor Raja, Muhammad Shoaib, Muhammed Shabab Alhothuali

**Affiliations:** 1 Department of Mathematics, Faculty of Science, King Abdulaziz University, Jeddah, 21589 Saudi Arabia; 2 Future Technology Research Center, National Yunlin University of Science and Technology, 123 University Road, Section 3, Douliou, Yunlin 64002 Taiwan, ROC; 3 Department of Mathematics, COMSATS University Islamabad, Attock Campus, Attock, 43600 Pakistan

**Keywords:** Mathematics and computing, Applied mathematics, Computational science

## Abstract

In the current study, a modern implementation of intelligent numerical computational solver introduced using the Levenberg Marquardt algorithm based trained neural networks (LMA-TNN) to analyze the wire coating system (WCS) for the elastic-viscous non-Newtonian Eyring–Powell fluid (EPF) with the impacts of Joule heating, magnetic parameter and heat transfer scenarios in the permeable medium. The nonlinear PDEs describing the WCS-EPF are converted into dimensionless nonlinear ODEs containing the heat and viscosity parameters. The reference data for the designed LMA-TNN is produced for various scenarios of WCS-EPF representing with porosity parameter, non-Newtonian parameter, heat transfer parameter and magnetic parameter for the proposed analysis using the state of the art explicit Runge–Kutta technique. The training, validation, and testing operations of LMA-TNN are carried out to obtain the numerical solution of WCS-EPF for various cases and their comparison with the approximate outcomes certifying the reasonable accuracy and precision of LMA-TNN approach. The outcomes of LMA-TNN solver in terms of state transition (ST) index, error-histograms (EH) illustration, mean square error, and regression (R) studies further established the worth for stochastic numerical solution of the WCS-EPF. The strong correlation between the suggested and the reference outcomes indicates the structure’s validity, for all four cases of WCS-EPF, fitting of the precision $10^{-5}$ to $10^{-9}$ is also accomplished.

## Introduction

**Figure 1:**
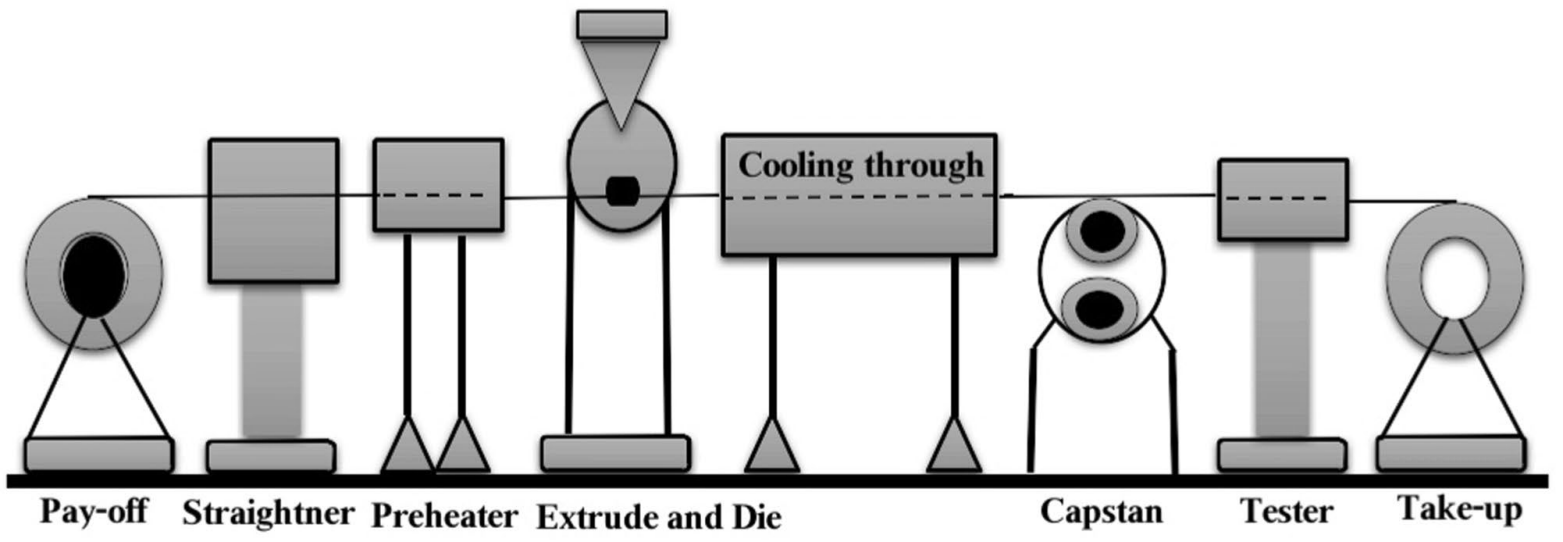
WCS-EPF for wire processing.

Wire coating covers an electrical conductor with a layer of dielectric material through a process called the Extrusion Process. This process is one of the most important and accurate production processes manufacturing the insulated wires and cables usually used in polymer melt industries. The system model operations for WCS-EPF are shown below in Fig. 1^1^. In this process, the coating material, which is in the shape of granules, is melted that introducing this material into the Extruder, where the high temperature and pressure are appropriate to meet the material and deliver it to the required state. Then the material reaches so-called Extruder Head, which guides the liquid material through the tip and dies to give the desired tube shape and the required thickness. Then the material comes out from the Head’s core to stick to the wire directly and form according to the wire’s shape. Immediately after that, the water-cooling stage comes inside a stream of water that the coated wire passes through for a certain distance and then dried with direct air. Then it is rolled onto a drum. The wire coated, regardless of its shape or content, by two types of separate dies, one in the form of a tube and the other pressure die like a ring. The flow through this mold is identical to the flux by the guttural area consisting of two molds, one is fixed externally, and the other internal is dynamic moving in the flow path. Depending on the die geometry, dynamic velocity, and heat of the wire and melt polymer, various kinds of liquids are used for wire coating.

The study of WCS in Newtonian fluids systems has attracted the research community with their broad interest. Fluidic systems are classified into two types depending on the viscosity: Newtonian fluids as water, honey, oil, and alcohol, and non-Newtonian fluids as butter, ketchup, mayonnaise, milk, and blood. The non-Newtonian fluidic systems with the variable viscosity havind wide range of applications in industry and fluid mechanics. Many researchers^1–4^ have used different non-Newtonian fluid models for materials of wire coating along with the affects of joule heating and hall current.A magnetic field provides an influencing source in the magnetic hydrodynamic (MHD) process and significantly impacts fluid movement in the wire coating processes. WCS analyses involving MHD have been extensively exploited by scholars in the latest years due to its widespread implementations in the industrial system, such as glazier processing and attractive-materials. Many researchers^5–7^ studied the MHD process with the impact of an applied magnetic field for the dynamics of WCS.

Owing to the broad reach in engineering science, fluid flow across a porous medium has unparalleled interest for researchers. Some common permeable media are timber, mineral foams, and crags of the carbonate, etc. Over time, the study about the application of heat transfer for WCS subjected to non-Newtonian fluids has acquired popularity owing to its application for different manufacturers. Rehman and Nadeem^8^ examined transfer of heating impact on the processing for the motion of multi-directional stagnation flow. MHD along with heating impacts on the WCS for various types of fluids are investigated by several researchers^9–16^. All these researches have so far introduced numerical/analytical of deterministic techniques for the solution of WCS subjected to different types of Newtonian and non-Newtonian fluids. In contrast, artificial intelligence-based numerical soft computing solver is relatively less discovered/exploited in the field of computing fluid mechanics problems, especially for WCS in different flow dynamics.

The approximate numerical solutions based on stochastic techniques are obtained primarily by modeling artificial neural networks (ANN) and optimizing them with both the mixture of global and local search approaches for solving a range of problems based on ordinary and partial differential systems. Recent applications of stochastic numerical computing solvers include nonlinear systems emerging in astrophysics^17, 18^, nanotechnologies^19^, fluid mechanics^20–24^, plasma physics^25, 26^, fuel catching fire model^27^, magneto-hydrodynamics^28, 29^, electrical conduction solids^30^, energy^31^, rotating electrical devices^32^, thermodynamics^33^, heat conduction^34^, electromagnetic^35^, bioinformatics^36^, and COVID-19 virus spread model^37–39^ are few important examples of such solutions. These facts motivate the researchers to explore and use soft-computing stochastic methods to create an effective, alternative, and feasible computing model for solving the fluid dynamics systems associated with the wire coating operation.

Throughout this research study, the innovative ideas about the proposed problem and soft computational model are illustrated as follows:

- A new implementation of intelligent computational system of the artificial intelligence is introduced by incorporating the solver LMA-TNN for interpreting the fluidic system WCS-EPF along with the impacts of Joule heating, applied magnetic field and transfer of heat in the permeable medium for different scenarios.
- The mathematical formulation is introduced with nonlinear differential equations systems for WCS-EPF, which are converted into dimensionless nonlinear ODEs representing the mathematical modeling of heat-based changing viscosity.
- A set of data for suggested LMA-TNN is produced for WCS-EPF on the in terms of physical quantities such as the porosity parameter, non-Newtonian parameter, magnetic parameter, heat transfer parameter utilizing the capability of explicit Runge–Kutta technique.
- The training, validation, and testing operations of the LMA-TNN are incorporating by designing the constant viscosity, Reynolds, and Vogel’s models for various scenarios and comparing the reference outcomes to confirm the accuracy of designed solver LMA-TNN.
- The suggested LMA-TNN performance to efficiently solve the WCS-EPF is further verified by using convergence graphs of the fitness-dependent mean square error, histogram error, and regression measure.

The detailed mathematical modeling of the wire coating with the heat-based changing viscosity using Vogel’s and Reynolds models have described in “Mathematical model” section. The solution methodology along with the results of the suggested LMA-TNN based on variants of WCS-EPF is briefly explained in “Methodology and discussions” section. The “Conclusion” section consists of the conclusion of the research study.

## Mathematical model

**Figure 2:**
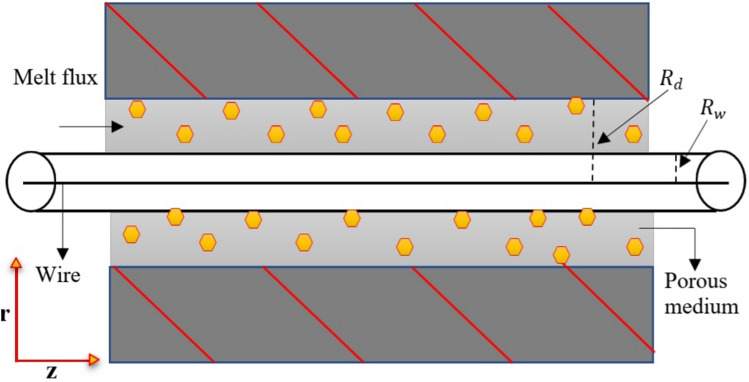
Graphical representation of fluid flow through a hydrodynamic pressure unit.

Figure 2 illustrates the structure of the study. Let $R_{d}$, be the radius, *L* is the length of die and the saturated temperature $\theta _{d}$ since the viscoelastic Eyring–Powell material is not compressible, therefore the wire temperature exceeds $\theta _{\text {w}}$, the radius is equivalent to $R_{\text {w}}$ and the velocity $U_{\text {w}}$ in the porous medium. After that, the wire is dragged across the center length in the fixed stress mold. The outflow liquid is concurrently dominated by the unified differential stress $\frac{\text {d} p}{\text {d} z}$ across the axial direction with the attractive strength $B_{o}$. The magnetic force is vertical along the path of the incompressible non-Newtonian Eyring–Powell fluid flow. We used the concept of low Reynolds number in our study to minimize or ignore the disturbance in the magnetic field.

For this study, the governing system of the suggested wire coating as follows^16^:

1 $$\left( \frac{1}{r}\right) \frac{d}{d r}\left( r\left\{ \left( \mu +\frac{1}{\beta c}\right) \frac{d w}{d r}-\frac{1}{6 \beta c^{3}}\left( \frac{d w}{d r}\right) ^{3}\right\} \right) -\frac{\mu w}{{K_{P}}^{\prime}}=0,$$

2 $$K\left( \frac{d^{2} \theta }{d r^{2}}+\frac{1}{r} \frac{d \theta }{d r}\right) +\left[ \left( \mu +\frac{1}{\beta c}\right) \frac{d w}{d r}-\frac{1}{6 \beta c^{3}}\left( \frac{d w}{d r}\right) ^{3}\right] \frac{d w}{d r}+Q_{o}\left( \theta -\theta _{w}\right) =0,$$

with boundary conditions

3 $$\begin{aligned} \quad \begin{aligned} w\left( R_{\text {w}}\right) =U_{\text {w}}, \quad w\left( R_{d}\right) =0, \quad \theta \left( R_{\text {w}}\right) =\theta _{\text {w}}, \quad \theta \left( R_{d}\right) =\theta _{d}, \end{aligned} \end{aligned}$$

where *w* and $\theta$ represent the velocity and temperature variables in the trend of $\vec {r}$, respectively, $Q_o$ represents the level of heat generation through volume, and *c* denotes the constant of matter.

Using the dimensionless variables and parameters, we get three mathematical models given in as follow:

Constant viscosity:

4 $$(1+n)\left[ r \frac{\text {d}^{2} u}{\text {d} r^{2}}+\frac{\text {d} u}{\text {d} r}\right] -\alpha \left[ \left( \frac{\text {d} u}{\text {d} r}\right) ^{3}+3 r\left( \frac{\text {d} u}{\text {d} r}\right) ^{2} \frac{\text {d}^{2} u}{\text {d} r^{2}}\right] -K_{\text {P}} u r=0,$$

5 $$\frac{d^{2} \theta }{d r^{2}}+\frac{1}{r} \frac{d \theta }{d r}+B_{r}(1+n)\left( \frac{d u}{d r}\right) ^{2}+\alpha B_{r}\left( \frac{d u}{d r}\right) ^{4}+Q \theta =0,$$

with the following boundary conditions

6 $$u(1)=1,\quad \theta (1)=0, \quad u(\delta )=0, \quad \theta (\delta )=1.$$

Vogel’s model:

7 $$\begin{aligned}&{\frac{\text {d}^{2} u}{\text {d} r^{2}}\left[ r \Omega \left( 1-\frac{D}{\left( B^{*}\right) ^{2}} \theta \right) +r n-3 r \alpha \left( \frac{\text {d} u}{\text {d} r}\right) ^{2}\right] +\frac{\text {d} u}{\text {d} r}\left[ \left( 1-\frac{D}{\left( B^{*}\right) ^{2}} \theta \right) \Omega +n- \frac{D}{\left( B^{*}\right) ^{2}} r\Omega \frac{\text {d} \theta }{\text {d} r}\right] }-\alpha \left( \frac{\text {d} u}{\text {d} r}\right) ^{3}\nonumber \\&\quad -K_{P} u r=0, \end{aligned}$$

8 $$\begin{aligned}&\frac{d^{2} \theta }{d r^{2}}+\frac{1}{r} \frac{d \theta }{d r}-\Omega B_{r}\left( \frac{D \theta }{\left( B^{*}\right) ^{2}}+1\right) \left( \frac{d u}{d r}\right) ^{2}+\left( \frac{d u}{d r}\right) ^{2}(n+\alpha )B_{r}+ Q \theta =0, \end{aligned}$$

with boundary conditions

9 $$u(1)=1,\quad \theta (1)=0, \quad u(\delta )=0, \quad \theta (\delta )=1.$$

Reynolds model:

10 $$\begin{aligned}&\frac{\text {d}^{2} u}{\text {d} r^{2}}\left[ r\left( 1-\beta _{n} m \theta \right) +r n-3 r \alpha \left( \frac{\text {d} u}{\text {d} r}\right) ^{2}\right] +\frac{\text {d} u}{\text {d} r}\left[ 1-\beta _{n} m \theta +n-\beta _{n} m r \frac{\text {d} \theta }{\text {d} r}\right] -\alpha \left( \frac{\text {d} u}{\text {d} r}\right) ^{3}\nonumber \\&\quad -K_{\text {P}} u r=0, \end{aligned}$$

11 $$\begin{aligned}&\frac{d^{2} \theta }{d r^{2}}+\frac{1}{r} \frac{d \theta }{d r}+\left( 1-\beta _{n} m \theta \right) B_{r}\left( \frac{d u}{d r}\right) ^{2}+B_{r}\left( \frac{d u}{d r}\right) ^{2}(n+\alpha )+Q \theta =0, \end{aligned}$$

with boundary conditions as

12 $$u(1)=1,\quad \theta (1)=0, \quad u(\delta )=0, \quad \theta (\delta )=1.$$

## Methodology and discussions

In artificial intelligence (AI), supervised machine learning relates to a category of algorithms and paradigms that describe a predictive model utilizing datasets with known outputs. The approach is learned via an effective teaching algorithm such as artificial neural networks that usually use optimization procedures to reduce error function.

**Figure 3:**
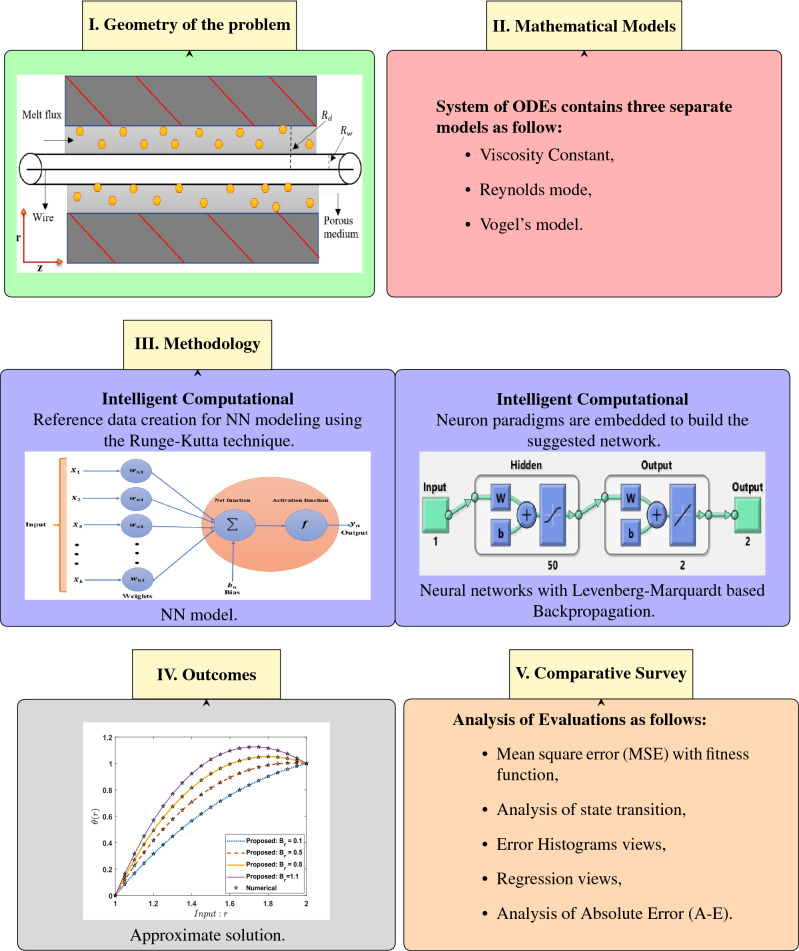
Scheme operation of the suggested LMA-TNN for the wire coating system.

**Figure 4:**
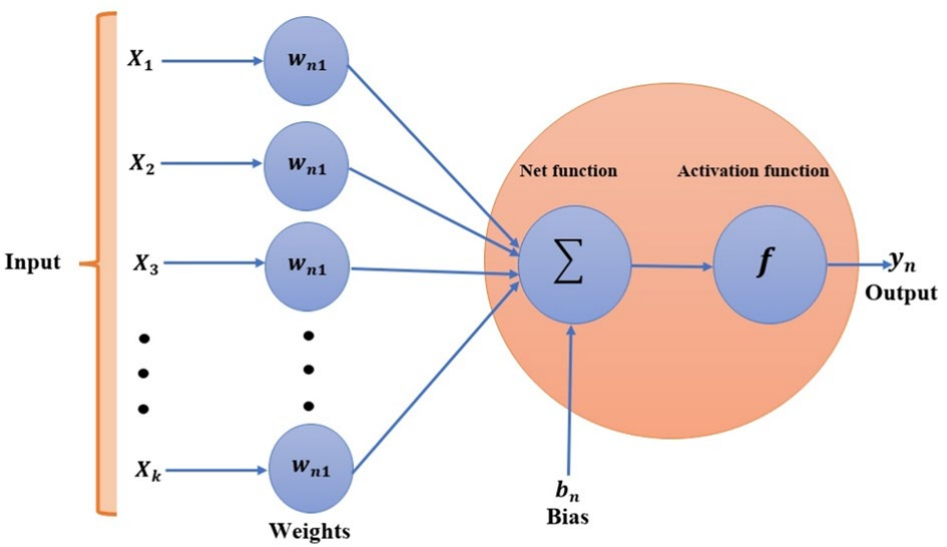
A single neural network model architecture.

Here, the technical solution involves two steps: the first part includes the required explanation for the design of LMA-TNN data sets; the second part describes the process for applying LMA-TNN. The complete typical procedure is shown in Fig. 3, whereas the suggested solver as a single neural paradigm is illustrated in Fig. 4. Numerical treatment with LMA-TNN is performed for the heat-based changing viscosity paradigm provided in Eqs. (4)–(12). The suggested LMA-TNN is introduced for different scenarios, i.e., S-1 to S-5 corresponding each case of Reynolds model whereas S-1 to S-4 corresponding each case of Constant Viscosity and Vogel’s models, as shown in Tables 1, 2 and 3 , respectively.

**Table 1:** Variants of WCS-EPF for constant viscosity.

Scenarios	Cases	Physical amounts of significance				
		$B_{r}$	*Q*	$K_{P}$	$\alpha$	*n*
(1) Variation in $B_{r}$	1	0.1	0.6	0.5	0.01	0.2
	2	0.5	0.6	0.5	0.01	0.2
	3	0.8	0.6	0.5	0.01	0.2
	4	1.1	0.6	0.5	0.01	0.2
(2) Variation in *Q*	1	0.1	0.5	0.5	1.5	0.2
	2	0.1	1.5	0.5	1.5	0.2
	3	0.1	2.5	0.5	1.5	0.2
	4	0.1	3.5	0.5	1.5	0.2
(3) Variation in $K_{P}$	1	0.2	0.7	0.1	1.5	0.2
	2	0.2	0.7	0.3	1.5	0.2
	3	0.2	0.7	0.7	1.5	0.2
	4	0.2	0.7	1	1.5	0.2
(4) Variation in $\alpha$	1	1.1	0.3	0.3	0.01	0.1
	2	1.1	0.3	0.3	0.9	0.1
	3	1.1	0.3	0.3	1.5	0.1
	4	1.1	0.3	0.3	2	0.1

**Table 2:** Variants of WCS-EPF for Vogel’s model.

Scenarios	Cases	Physical amounts of significance						
		$B_{r}$	*Q*	*D*	$\Omega$	$K_{P}$	$B^{`}$	n
(1) Variation in $B_{r}$	1	0.3	0.3	0.6	1	0.2	1	0.3
	2	0.5	0.3	0.6	1	0.2	1	0.3
	3	0.7	0.3	0.6	1	0.2	1	0.3
	4	0.9	0.3	0.6	1	0.2	1	0.3
(2) Variation in *Q*	1	0.2	0.1	0.6	1	0.2	1	0.3
	2	0.2	0.5	0.6	1	0.2	1	0.3
	3	0.2	1	0.6	1	0.2	1	0.3
	4	0.2	1.5	0.6	1	0.2	1	0.3
(3) Variation in *D*	1	0.6	0.3	0.4	1	0.4	0.3	0.5
	2	0.6	0.3	0.6	1	0.4	0.3	0.5
	3	0.6	0.3	0.8	1	0.4	0.3	0.5
	4	0.6	0.3	1	1	0.4	0.3	0.5
(4) Variation in $\Omega$	1	0.3	0.5	0.5	0.1	0.5	0.3	0.4
	2	0.3	0.5	0.5	0.7	0.5	0.3	0.4
	3	0.3	0.5	0.5	1.2	0.5	0.3	0.4
	4	0.3	0.5	0.5	1.5	0.5	0.3	0.4

**Table 3:** Variants of WCS-EPF for Reynolds model.

Scenarios	Cases	Physical amounts of significance						
		$B_{r}$	*Q*	*n*	$K_{P}$	$\alpha$	$\beta _{n}$	*m*
(1) Variation in $B_{r}$	1	0.3	0.2	0.1	0.1	0.9	0.1	0.1
	2	0.5	0.2	0.1	0.1	0.9	0.1	0.1
	3	0.7	0.2	0.1	0.1	0.9	0.1	0.1
	4	0.9	0.2	0.1	0.1	0.9	0.1	0.1
(2) Variation in *Q*	1	0.5	0.5	1.2	0.1	0.2	2.1	0.2
	2	0.5	1	1.2	0.1	0.2	2.1	0.2
	3	0.5	1.5	1.2	0.1	0.2	2.1	0.2
	4	0.5	2.5	1.2	0.1	0.2	2.1	0.2
(3) Variation in *n*	1	0.1	0.3	4	0.2	0.3	1.2	0.13
	2	0.1	0.3	6	0.2	0.3	1.2	0.13
	3	0.1	0.3	8	0.2	0.3	1.2	0.13
	4	0.1	0.3	10	0.2	0.3	1.2	0.13
(4) Variation in $K_{P}$	1	0.1	0.2	0.1	0.3	1	0.1	0.4
	2	0.1	0.2	0.1	0.5	1	0.1	0.4
	3	0.1	0.2	0.1	0.7	1	0.1	0.4
	4	0.1	0.2	0.1	0.9	1	0.1	0.4
(5) Variation in $\alpha$	1	0.5	0.1	0.3	0.4	0.1	1.2	0.1
	2	0.5	0.1	0.3	0.4	0.4	1.2	0.1
	3	0.5	0.1	0.3	0.4	0.7	1.2	0.1
	4	0.5	0.1	0.3	0.4	1	1.2	0.1

The reference data for LMA-TNN is obtained by employing the Runge–Kutta technique with the help of NDSolver in Mathematica. The suggested LMA-TNN is employed in the form of data sets; the output toward a single input is integrated with the assist of ’nftool’ in the toolbox of the neural network through MATLAB software (R2020b Update 5 (9.9.0.1592791), https://www.mathworks.com/academia/tah-portal/king-abdulaziz-university-40773215.html). Total 1001 given data points for each variable (*u*(*r*), $\theta (r)$) is determined between 1 and $\delta$ = 2 by keeping the step size 0.001. Then this data is divided into three datasets: the testing, the validation, and the training, in different proportions to determine the percentage that gives a better convergence. These datasets have been generated by reference standard solutions for the system of non-linear higher-order differential equations interpreting the elastic-viscous Eyring–Powell fluidic system WCS-EPF along with the impacts of Joule heating, applied magnetic field, and transfer of heat in the permeable medium for different scenarios. The training, validation, and testing processes for Levenberg–Marquardt backpropagation neural networks are divided as follow:

- 80$\%$ of the dataset are assigned for the training.
- 10$\%$ of the dataset are assigned for the validation.
- 10$\%$ of the dataset are assigned for the testing.

**Figure 5:**
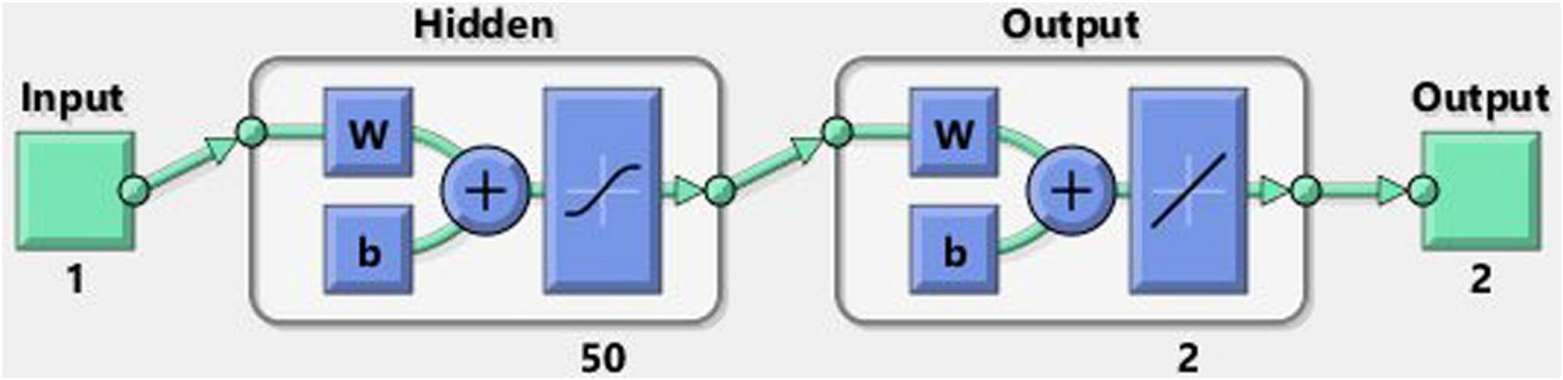
Design of suggested neural networks.

After performing several tests to obtain optimum measurement accuracy using some hidden neurons, the best is 50 neurons, whereas training the weights of neural networks requires the Levenberg Marquardt based back-propagation. The design of the suggested network can be seen in Fig. 5.

**Figure 6:**
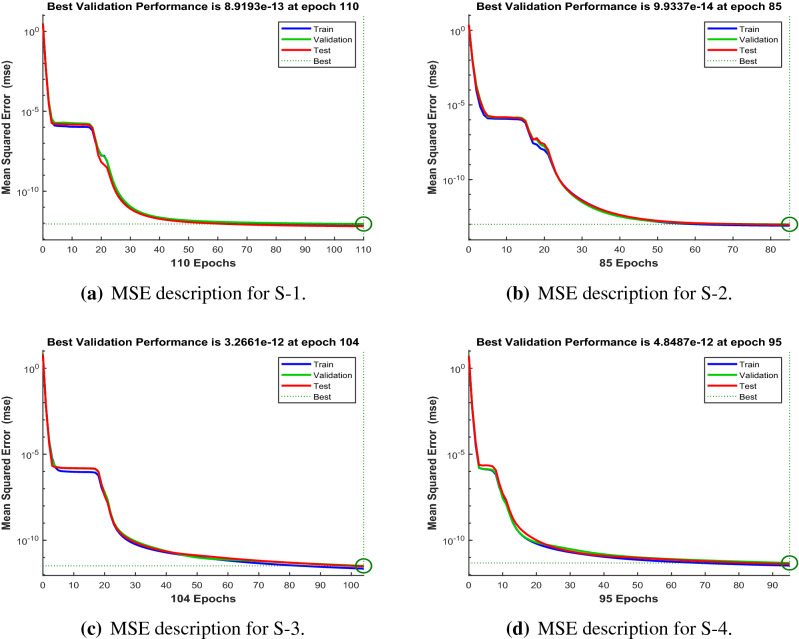
Performance of the LMA-TNN for testing, validation, and training procedures of Case 2 in constant viscosity.

**Figure 7:**
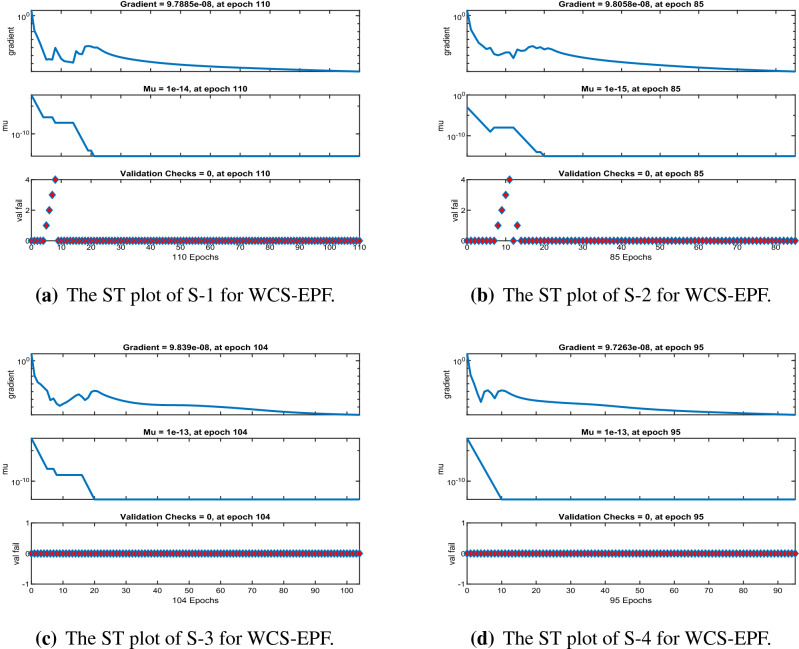
The state transition (ST) for Case2 in constant viscosity.

**Figure 8:**
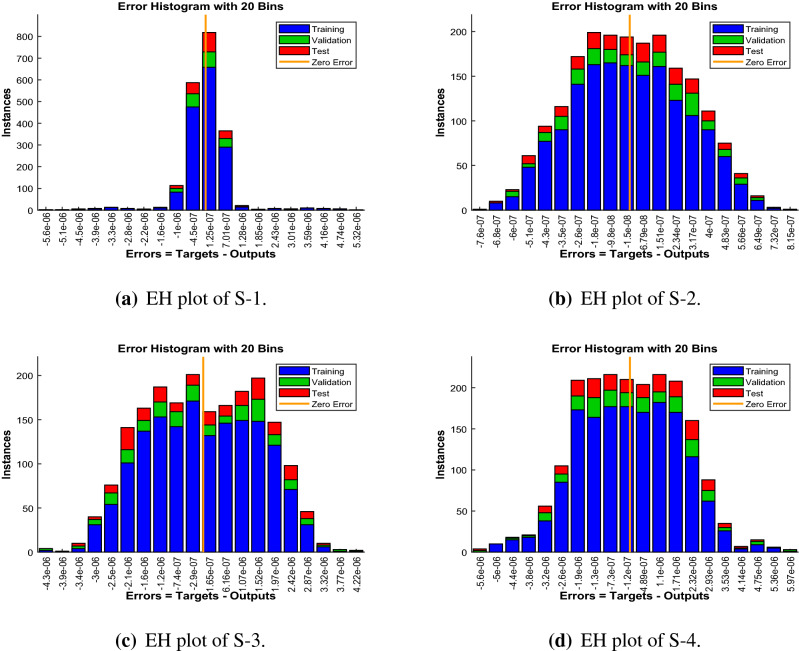
Error-histogram (EH) views of LMA-TNN for case 2 in constant viscosity.

**Figure 9:**
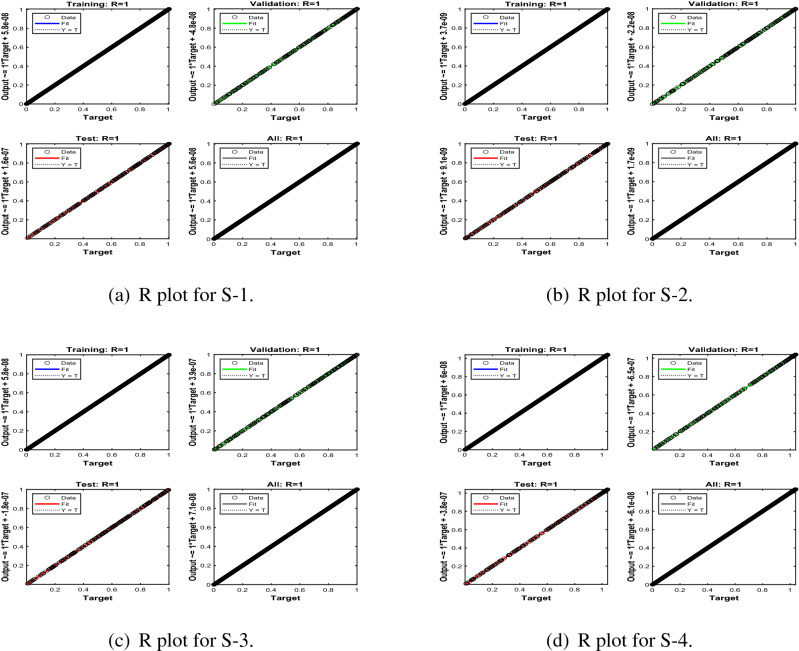
Regression (R) analysis of LMA-TNN for Case 2 in constant viscosity.

**Figure 10:**
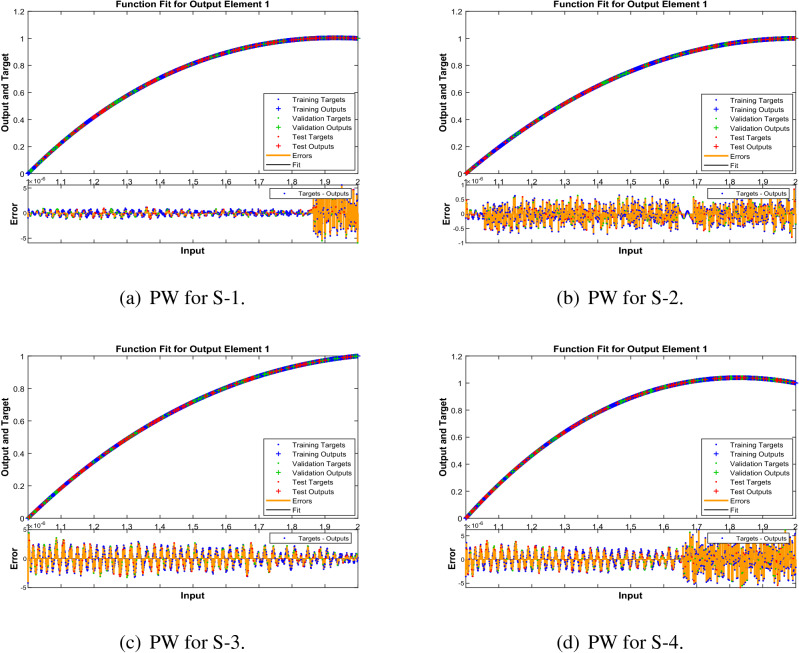
Reference solution comparison with LMA-TNN based performance view (PW) for case 2 in constant viscosity.

**Figure 11:**
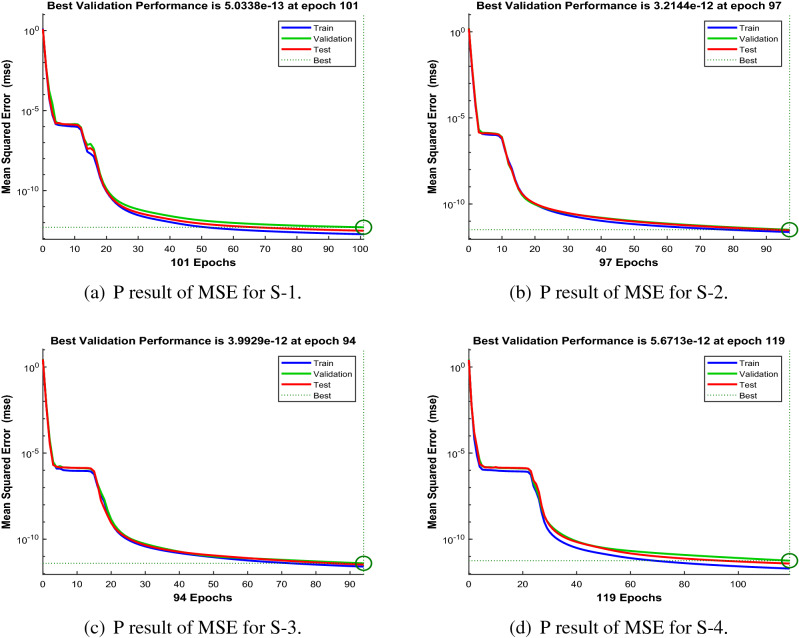
Performance (P) of the LMA-TNN for testing, validation, and training procedures of Case 2 in Vogel’s model.

**Figure 12:**
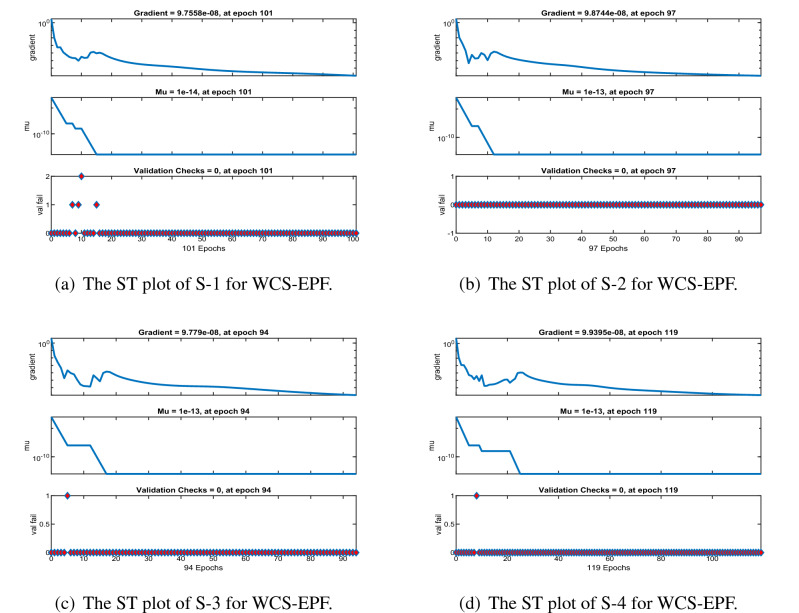
The state transition (ST) for Case 2 in Vogel’s model.

**Figure 13:**
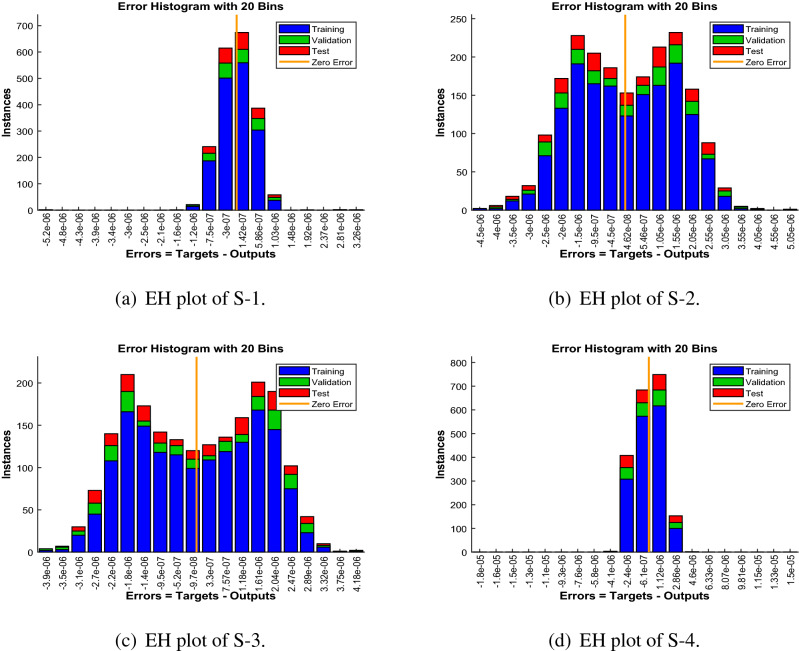
Error-histogram (EH) views of LMA-TNN for Case 2 in Vogel’s model.

**Figure 14:**
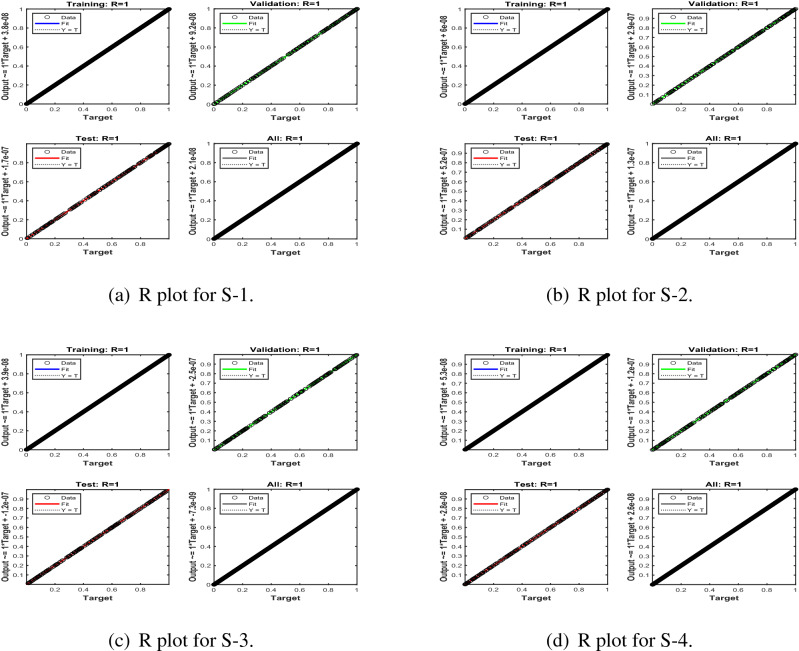
Regression (R) views of LMA-TNN results for Case 2 in Vogel’s model.

**Figure 15:**
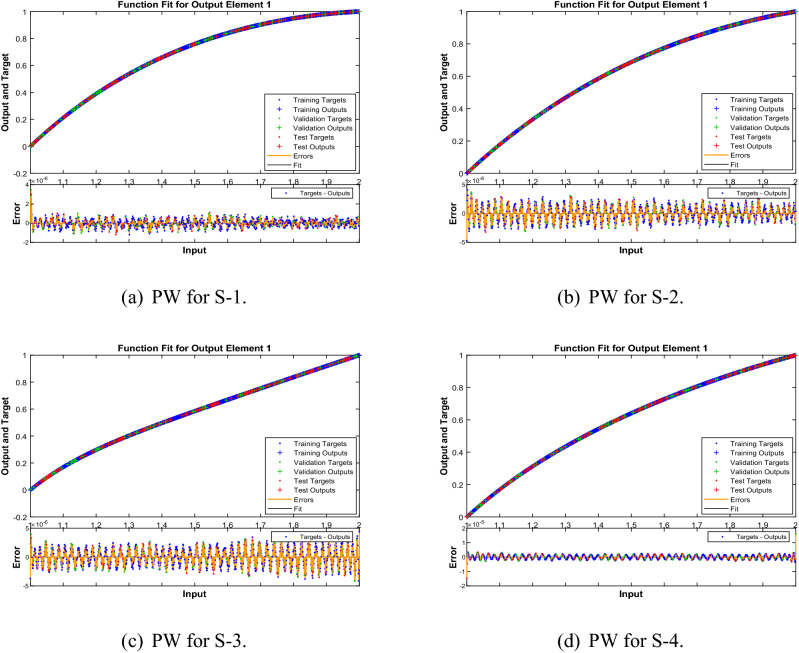
Reference solution comparison with LMA-TNN based performance view (PW) in Vogel’s model.

**Figure 16:**
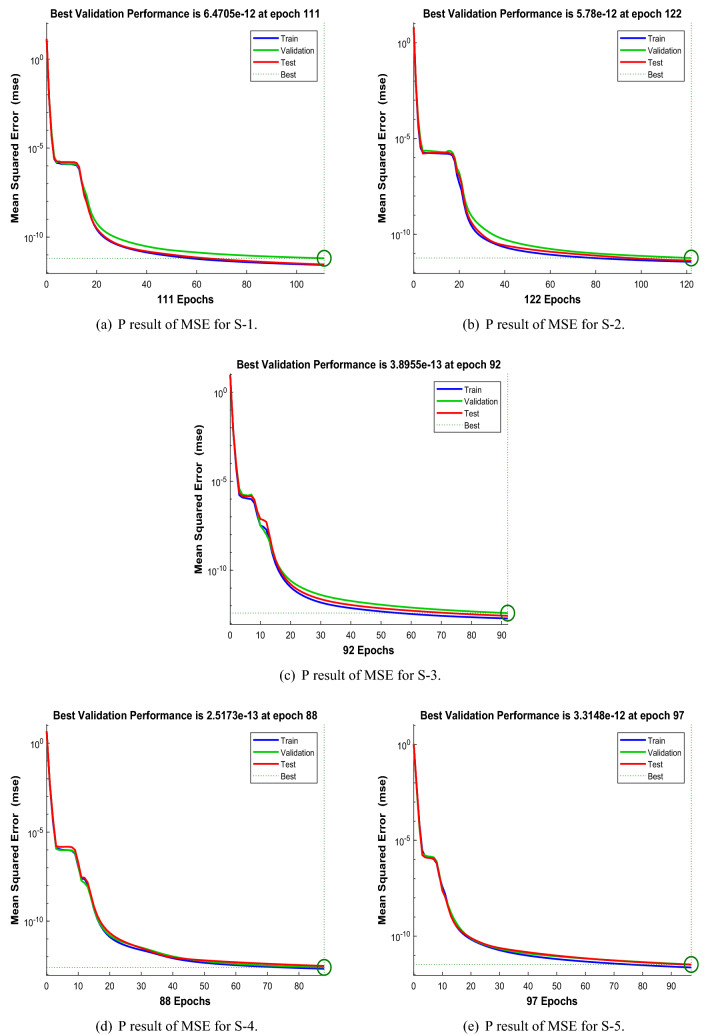
Performance (P) of the LMA-TNN for testing, validation, and training procedures of Case 4 in Reynolds model.

**Figure 17:**
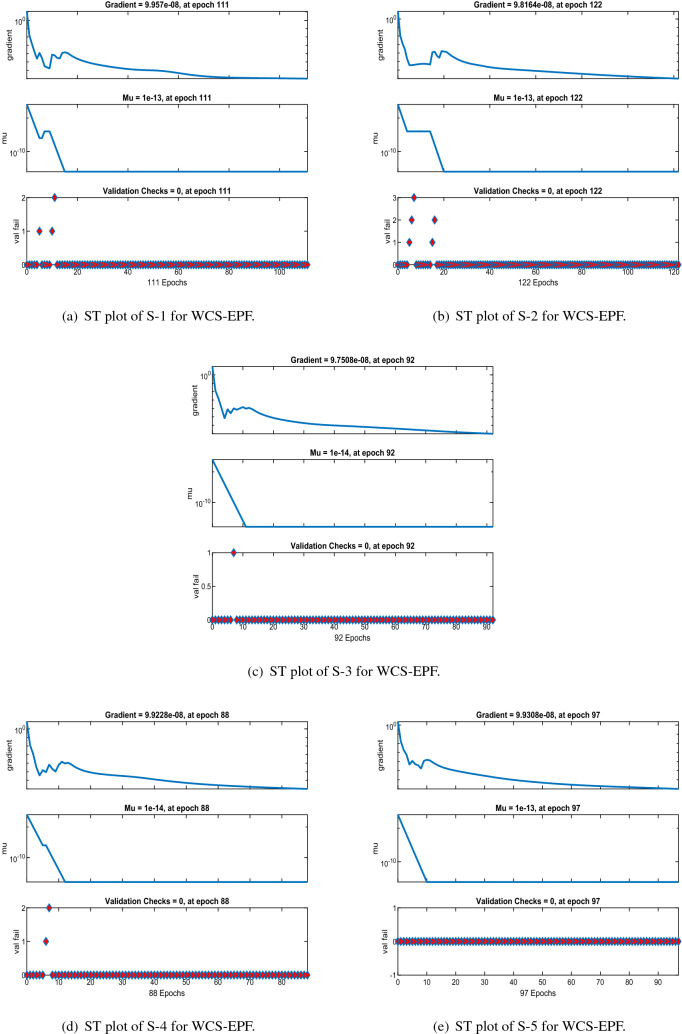
The State transition (ST) for Case-4 in Reynolds model.

**Figure 18:**
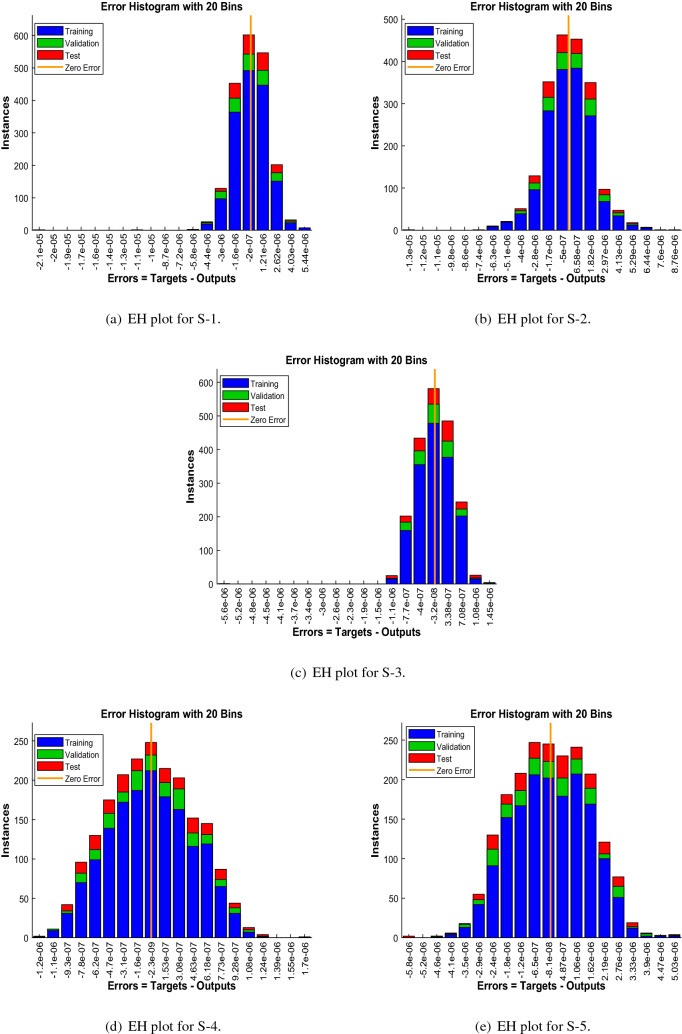
Error-histogram (EH) representation of LMA-TNN for Case 4 in Reynolds model.

**Figure 19:**
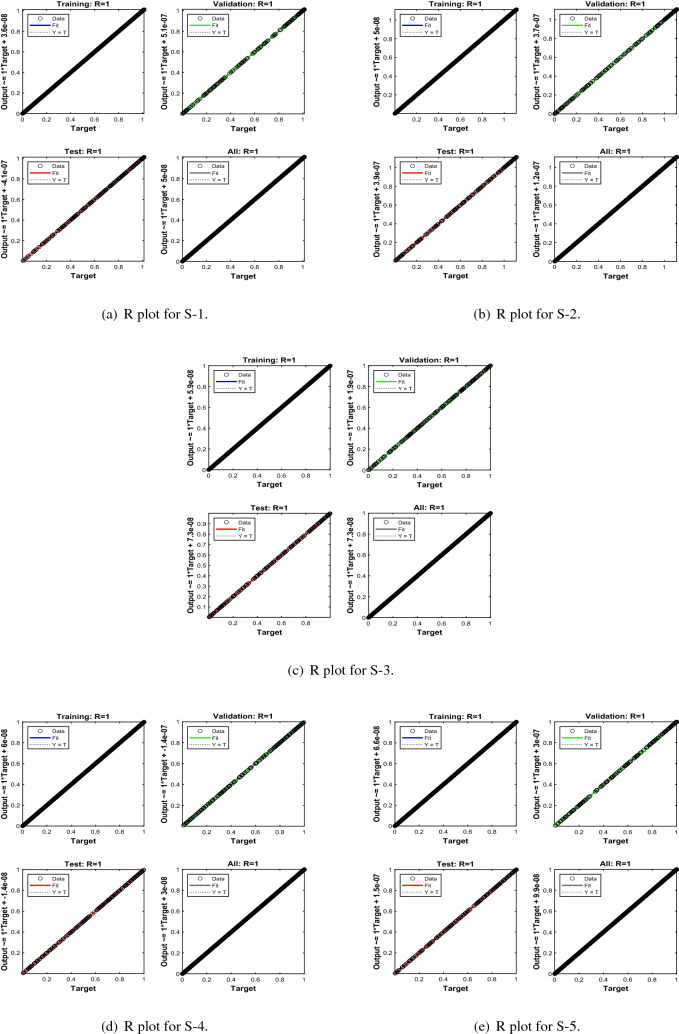
Regression (R) views of LMA-TNN results for Case 4 in Reynolds model.

**Figure 20:**
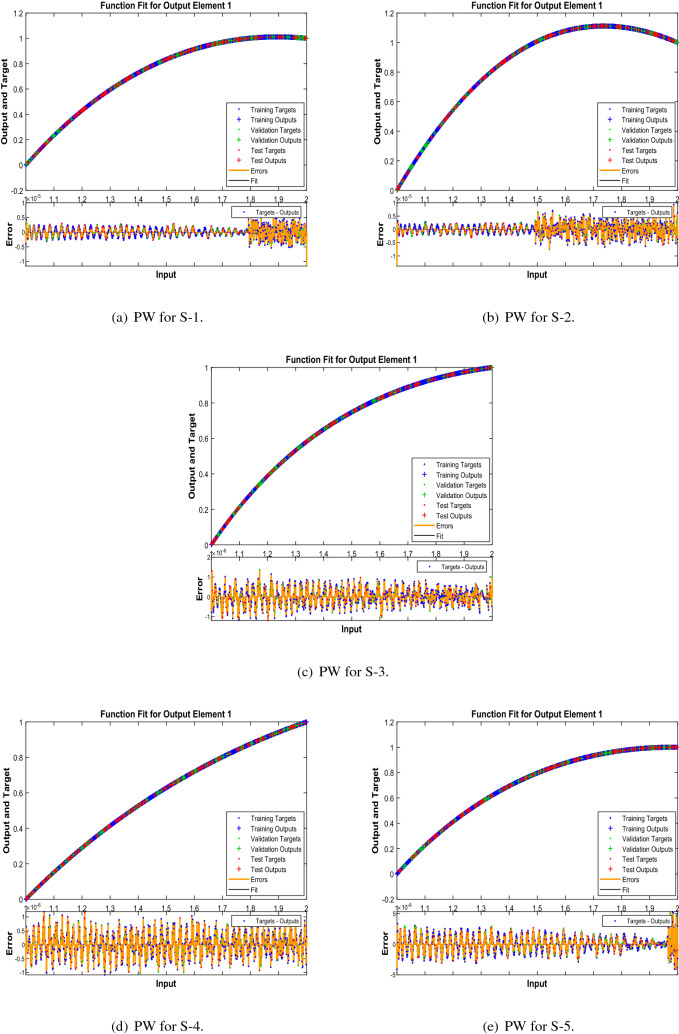
Reference solution comparison with LMA-TNN based performance view (PW) for case 4 in Reynolds model.

As shown in the above figure, the best artificial neural network structure for the data under study is (1 50 2), i.e., one input layer and a hidden layer containing 50 processing elements with two outputs. Using the neural network under supervision, the set of data consisting of 1001 points for each output treated according to different scenarios of all four cases, as shown by Tables 1, 2 and 3. The efficiency and precision investigation of the LMA-TNN process for All the scenarios of cases 2, 2, and 4 in Constant Viscosity, Vogel’s, and Reynolds models, respectively, is achieved graphically in Figs. 6, 7, 8, 9, 10, 11, 12, 13, 14, 15, 16, 17, 18, 19 and 20. The comparison of all numerical and random data that contains performance, Gradient, Mu, epochs, time, and mean squared error for testing, validation, and training for all four cases of every scenario as shown in Tables 4, 5 and 6.

Figures 6, 11 and 16 describe the mean squared error (MSE) based on training, testing, and validation operations for all scenarios in Constant Viscosity, Vogel’s and Reynolds models, respectively, for measuring neural network performance for predicting and relying on results while ensuring predictive accuracy. As in Fig. 6a–d, it clear that have the best accuracy and performance along with MSE about ($10^{-12}$, $10^{-13}$, $10^{-11}$ to $10^{-12}$, and $10^{-11}$ to $10^{-12}$) at 110, 85, 104, and 95 epochs, respectively, Also Fig. 11a–d display the MSE is about ($10^{-12}$ to $10^{-13}$, $10^{-11}$ to $10^{-12}$, $10^{-11}$ to $10^{-12}$, and $10^{-11}$ to $10^{-12}$) at 101, 97, 94, and 119 epochs, respectively and Fig. 16a–e show the MSE is about ($10^{-11}$ to $10^{-12}$, $10^{-11}$ to $10^{-12}$, $10^{-12}$ to $10^{-13}$, $10^{-12}$ to $10^{-13}$ and $10^{-11}$ to $10^{-12}$) at 111, 122, 92, 88 and 97 epochs, respectively. Fig. 7a–d explain the particular values of Gradient and Mu case 2 against four scenarios in Constant Viscosity are about ($9.79\times 10^{-08}$, $9.81\times 10^{-08}$, $9.84\times 10^{-08}$, and $9.73\times 10^{-08}$) and ($10^{-14}$, $10^{-15}$, $10^{-13}$, and $10^{-13}$), while the particular values of Gradient and Mu of case 2 for four scenarios in Vogel’s model are about ($9.76\times 10^{-08}$, $9.87\times 10^{-08}$, $9.78\times 10^{-08}$, and $9.94\times 10^{-08}$) and ($10^{-14}$, $10^{-13}$, $10^{-13}$, and $10^{-13}$) as seen in Fig. 12a–d. Moreover, Fig. 17a–e display the Gradient and Mu of case 4 for five scenarios in Reynolds model are about ($9.96\times 10^{-08}$, $9.82\times 10^{-08}$, $9.75\times 10^{-08}$, $9.92\times 10^{-08}$, and $9.93\times 10^{-08}$) and ($10^{-13}$, $10^{-13}$, $10^{-14}$, $10^{-14}$, and $10^{-13}$).

Error-histograms further analyze the error dynamics for every input data, and the outputs are shown in Figs. 8, 13 and 18 for the proposed problem WCS-EPF. The zero axis along with error box for all four scenarios in case 2 of Constant Viscosity is about $1.25\times 10^{-07}$, $-\,1.5\times 10^{-08}$, $1.65\times 10^{-07}$, and $-\,1.2\times 10^{-07}$, as shown in Fig. 8a–d, respectively. Whereas, Fig. 13a–d display the zero axis along with error box of reference for all four scenarios in case 2 of Vogel’s model is about $-\,2\times 10^{-07}$, $-5\times 10^{-07}$, $-\,3.2\times 10^{-08}$, $-\,2.3\times 10^{-09}$, and $-\,8.1\times 10^{-08}$. Besides, Fig. 18a–e illustrate the zero axis along with error box of reference is about $1.42\times 10^{-07}$, $4.62\times 10^{-08}$, $-\,9.7\times 10^{-08}$, and $-\,6.1\times 10^{-07}$ of case 4 for all five scenarios in Reynolds model. To judge the efficiency of training, validation, and test, from Figs. 9, 14 and 19 , it clear that the correlation function of the resulting errors indicates the efficiency of training the network, which is confirmed by the correlation coefficient R = 1, which illustrates the precision of LMA-TNN to solve the WCS-EPF.

In Figs. 10, 15 and 20 , comparative study based on the performance of outputs produced by LMA-TNN with numerical reference outcomes of the Runge-Kutta technique for the WCS-EPF, along with the dynamics of input error between 1 and 2 with step-size 0.001. Figures 10a–d show the maximal error achieved by the suggested LMA-TNN for validation, train, and test data is less than $5\times 10^{-6}$ for all four scenarios in case 2 of Constant Viscosity. While, the maximal error in Fig. 15a–d is less than $4\times 10^{-6}$, $5\times 10^{-6}$, $5\times 10^{-6}$, and $2\times 10^{-5}$ for all four scenarios in in case 2 of Vogel’s model. Also, in Fig. 20a–e, it clear that the maximal error is less than $5\times 10^{-5}$, $5\times 10^{-5}$, $2\times 10^{-6}$, $1\times 10^{-6}$, and $5\times 10^{-6}$ of case 4 for all five scenarios in Reynolds model.

The outcomes of WCS-EPF in the non-Newtonian flow by the solver LMA-TNN for solving every case of different scenarios are listed in Tables 4, 5 and 6. The performance of LMA-TNN is about $10^{-12}$ to $10^{-13}$, $10^{-12}$ to $10^{-14}$, $10^{-12}$, and $10^{-12}$ for all four scenarios of Constant Viscosity, respectively, as shown in Table 4. Moreover, the Table 5 illustrates performance of LMA-TNN is about $10^{-12}$ to $10^{-13}$, $10^{-12}$, $10^{-12}$, and $10^{-12}$ for all four scenarios of Vogel’s model, respectively and Table 6 provides the performance of LMA-TNN for all five scenarios of Reynolds model is about $10^{-12}$, $10^{-12}$, $10^{-13}$ to $10^{-12}$, $10^{-12}$ to $10^{-13}$, and $10^{-13}$ to $10^{-12}$, respectively. These outcomes show the harmonious performance of proposed solver LMA-TNN for the analysis of WCS-EPF.

**Figure 21:**
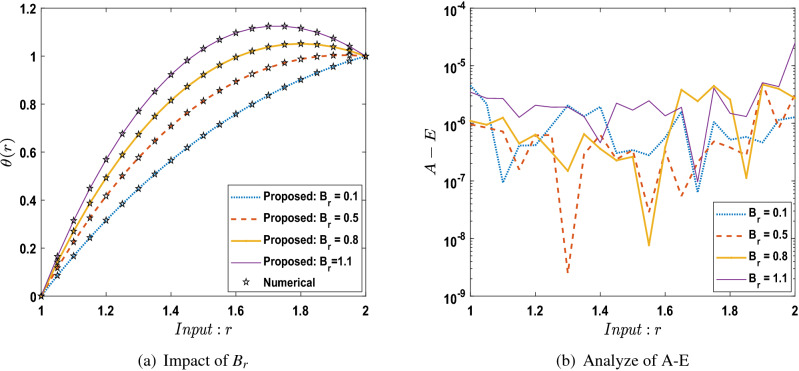
Comparison between LMA-TNN with results of numerical reference for scenario 1 in constant viscosity.

**Figure 22:**
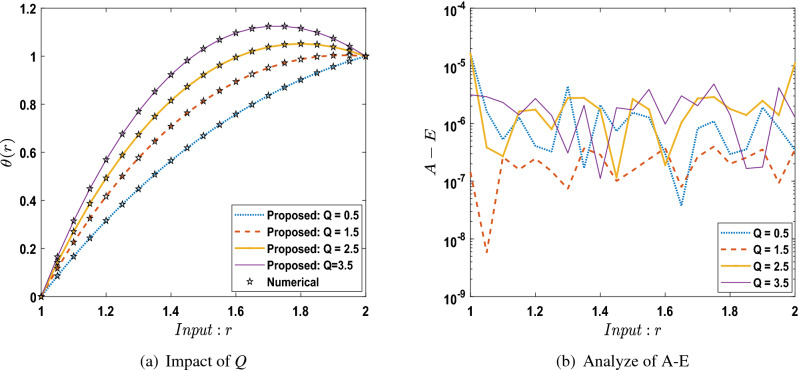
Comparison between LMA-TNN with results of numerical reference for scenario 2 in constant viscosity.

**Figure 23:**
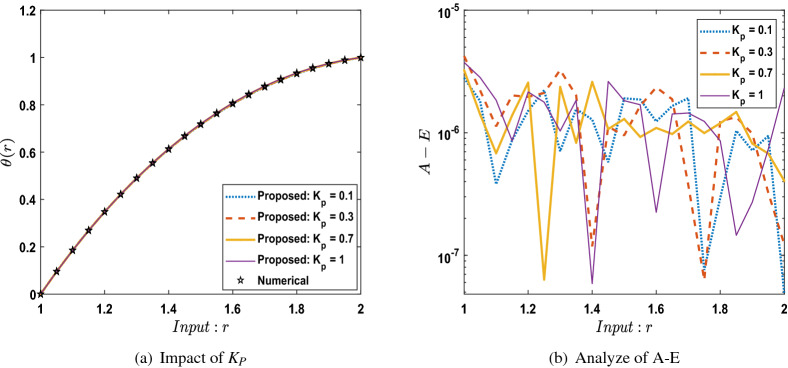
Comparison between LMA-TNN with results of numerical reference for scenario 3 in constant viscosity.

**Figure 24:**
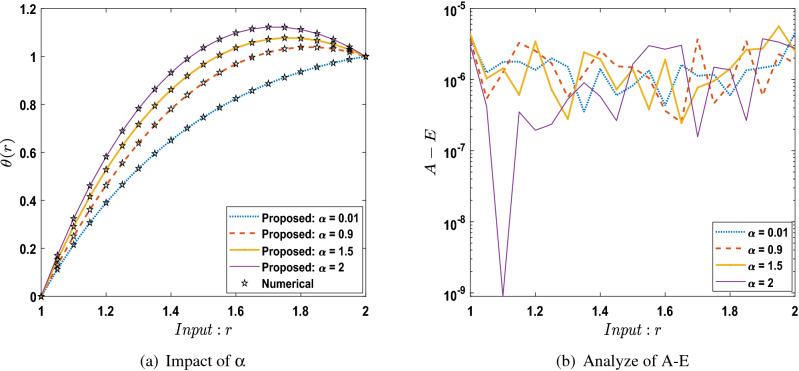
Comparison between LMA-TNN with results of numerical reference for scenario 4 in constant viscosity.

**Figure 25:**
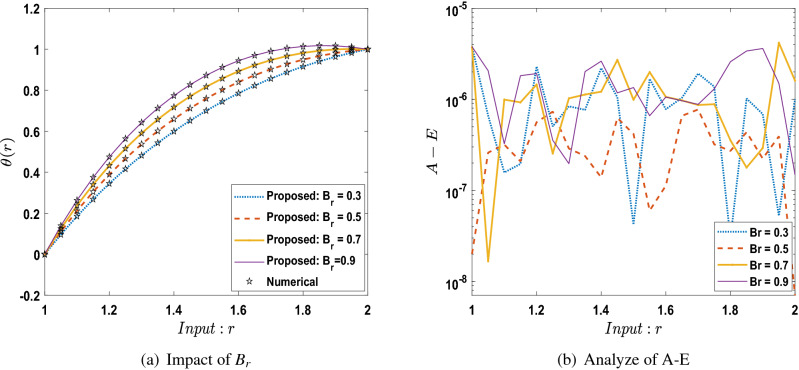
Comparison between suggested LMA-TNN with results of numerical reference for scenario 1 in Vogel’s model.

**Figure 26:**
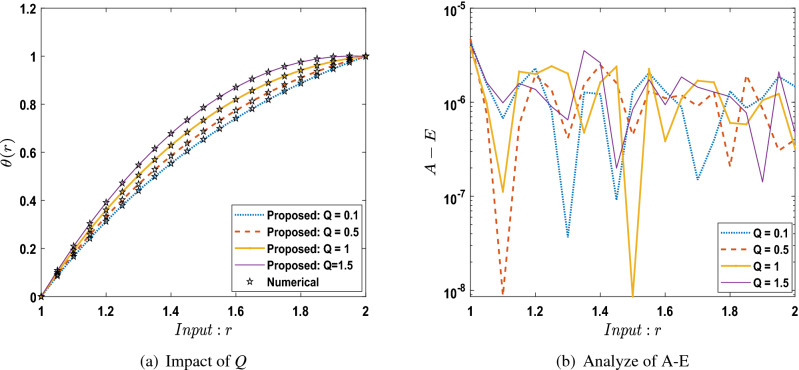
Comparison between suggested LMA-TNN with results of numerical reference for scenario 2 in Vogel’s model.

**Figure 27:**
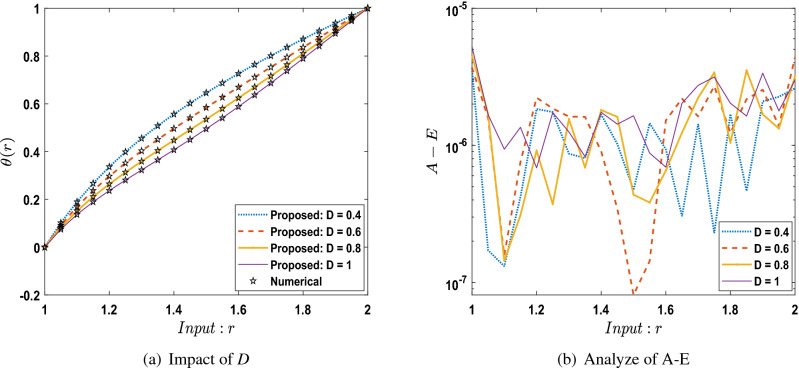
Comparison between suggested LMA-TNN with results of numerical reference for scenario 3 in Vogel’s model.

**Figure 28:**
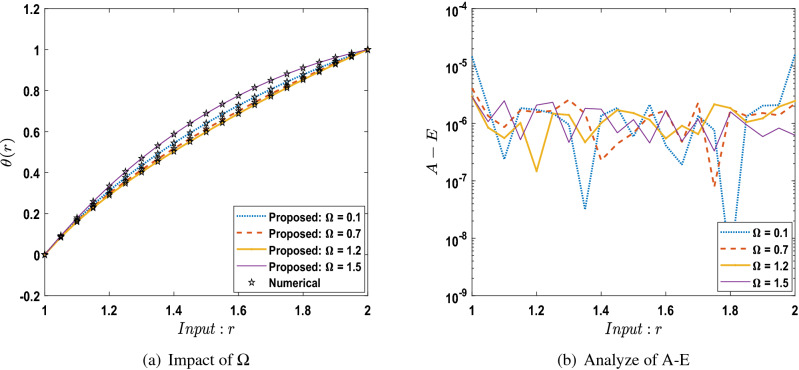
Comparison between suggested LMA-TNN with results of numerical reference for scenario 4 in Vogel’s model.

**Figure 29:**
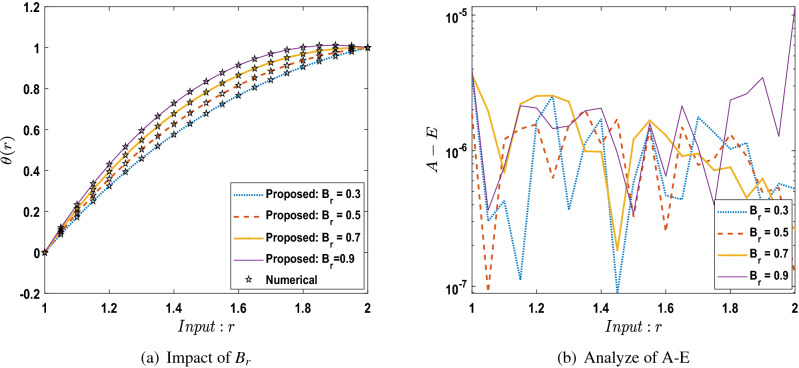
Comparison between suggested LMA-TNN with results of numerical reference for scenario 1 in Reynolds model.

**Figure 30:**
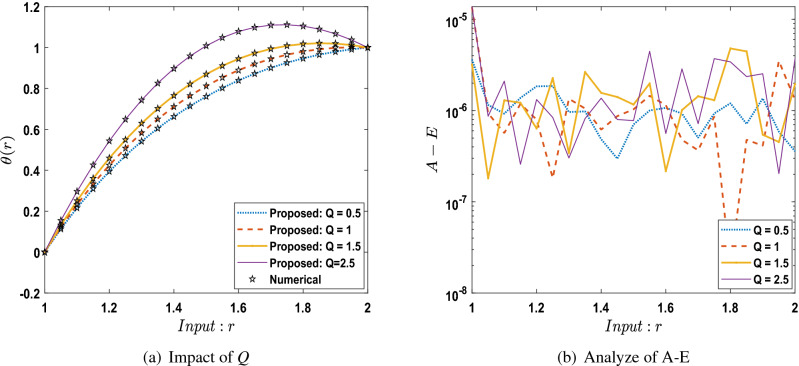
Comparison between suggested LMA-TNN with results of numerical reference for scenario 2 in Reynolds model.

**Figure 31:**
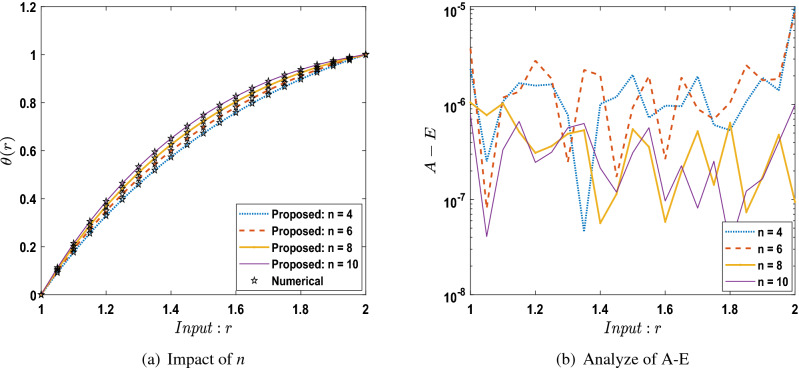
Comparison between suggested LMA-TNN with results of numerical reference for scenario 3 in Reynolds model.

**Figure 32:**
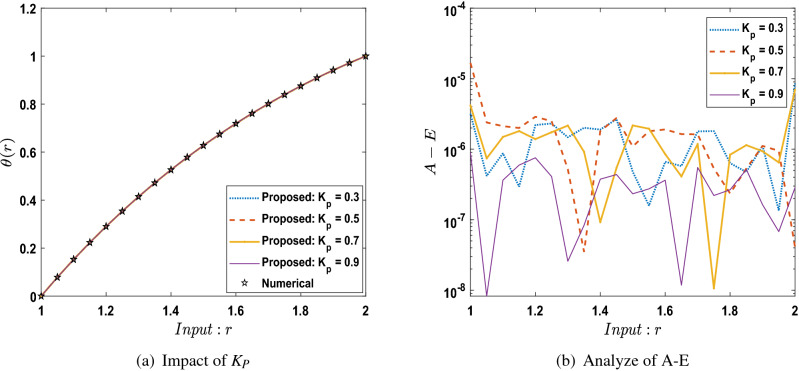
Comparison between suggested LMA-TNN with results of numerical reference for scenario 4 in Reynolds model.

**Figure 33:**
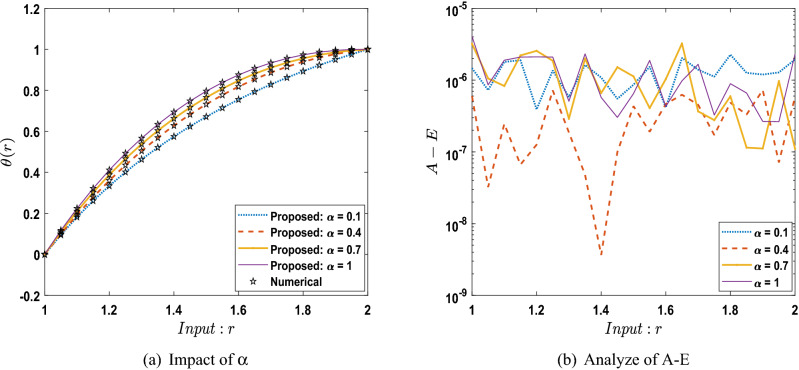
Comparison between suggested LMA-TNN with results of numerical reference for scenario 5 in Reynolds model.

The outcomes obtained by LMA-TNN are analyzed for the temperature distribution $\theta (r)$ for different scenarios based on parameter of interest, as shown in Figs. 21, 22, 23, 24, 25, 26, 27, 28, 29, 30, 31, 32 and 33. The outcomes of the solver LMA-TNN corresponding to the numerical solutions of the Runge–Kutta method for every scenario. Thus absolute error from the reference solutions is calculated to reach the accuracy criteria. In Figs. 21b, 22b, 23b and 24b indicate the absolute error(AE) is about $10^{-9}$ to $10^{-5}$, $10^{-8}$ to $10^{-5}$, $10^{-7}$ to $10^{-5}$, and $10^{-9}$ to $10^{-5}$ for S-1 , S-2, S-3, and S-4, respectively of Constant Viscosity. while, Figs. 25b, 26, 27 and 28b observe that AE is about $10^{-8}$ to $10^{-5}$, $10^{-8}$ to $10^{-5}$, $10^{-7}$ to $10^{-5}$, and $10^{-8}$ to $10^{-5}$ for S-1 , S-2, S-3, and S-4, respectively of Vogel’s model. In addition the AE is about $10^{-7}$ to $10^{-5}$, $10^{-8}$ to $10^{-5}$, $10^{-8}$ to $10^{-5}$, $10^{-8}$ to $10^{-5}$, and $10^{-9}$ to $10^{-5}$ for S-1 , S-2, S-3, S-4, and S-5, respectively of Reynolds model as shown in Figs. 29b, 30, 31, 32 and 33b. Numerical and graphical diagrams show the performance measures based on better precision, convergence analysis for the proposed computational technique LMA-TNN used to solve the impacts of WCS-EPF in the field of fluid dynamics.

**Table 4:** Total numerical analysis of LMA-TNN for constant viscosity.

Scenarios	Cases	MSE			Performance	Grad	Mu	Epochs	Time
		Training	Validation	Testing					
(1)	1	1.97E−12	2.54E−12	2.59E−12	2.54E−12	9.95E−08	1E−13	111	0.02
	2	7.79E−13	8.92E−13	6.41E−13	8.92E−13	9.79E−08	1E−14	110	0.01
	3	1.69E−12	1.8E−12	1.73E−12	1.8E−12	9.992E−08	1E−14	90	0.01
	4	4.08E−12	4.99E−12	5.54E−12	4.99E−12	9.999E−08	1E−13	104	0.02
(2)	1	2.12E−12	5.29E−12	2.74E−12	5.29E−12	9.71E−08	1E−13	107	0.02
	2	8.03E−14	9.93E−14	9.28E−14	9.93E−14	9.81E−08	1E−15	85	0.02
	3	3.21E−12	4.36E−12	8.87E−12	4.36E−12	9.91E−08	1E−13	105	0.02
	4	3.53E−12	5.27E−12	5.14E−12	5.27E−12	9.93E−08	1E−13	115	0.02
(3)	1	2.01E−12	2.37E−12	2.46E−12	2.37E−12	9.75E−08	1E−13	110	0.02
	2	2.26E−12	3.27E−12	3.06E−12	3.27E−12	9.9949E−08	1E−13	104	0.01
	3	1.78E−12	2.63E−12	2.39E−12	2.63E−12	9.90E−08	1E−13	123	0.02
	4	2.16E−12	5.77E−12	3.15E−12	5.77E−12	9.86E−08	1E−13	109	0.02
(4)	1	1.84E−12	2.63E−12	3.84E−12	2.63E−12	9.99E−08	1E−13	120	0.02
	2	3.40E−12	4.85E−12	4.13E−12	4.85E−12	9.73E−08	1E−13	95	0.01
	3	4.60E−12	6.76E−12	6.62E−12	6.76E−12	9.98E−08	1E−13	97	0.01
	4	2.02E−12	2.50E−12	2.76E−12	2.50E−12	9.45E−08	1E−14	110	0.02

**Table 5:** Complete numerical study of LMA-TNN for Vogel’s model.

Scenarios	Cases	MSE			Performance	Grad	Mu	Epochs	Time
		Training	Validation	Testing					
(1)	1	2.28E−12	3.73E−12	3.04E−12	3.73E−12	9.79E−08	1E−13	106	0.02
	2	1.89E−13	5.03E−13	3.07E−13	5.03E−13	9.76E−08	1E−14	101	0.01
	3	2.39E−12	3.46E−12	3.06E−12	3.46E−12	9.97E−08	1E−13	105	0.01
	4	3.08E−12	4.35E−12	3.71E−12	4.35E−12	9.89E−08	1E−13	106	0.01
(2)	1	2.10E−12	2.67E−12	2.96E−12	2.67E−12	9.91E−08	1E−13	104	0.02
	2	2.35E−12	3.21E−12	2.98E−12	3.21E−12	9.87E−08	1E−13	97	0.02
	3	2.25E−12	2.92E−12	2.67E−12	2.92E−12	9.86E−08	1E−13	100	0.02
	4	2.74E−12	3.38E−12	4.24E−12	3.38E−12	9.95E−08	1E−13	100	0.02
(3)	1	1.63E−12	2.07E−12	1.93E−12	2.07E−12	9.99E−08	1E−13	122	0.02
	2	2.56E−12	3.993E−12	3.36E−12	3.993E−12	9.78E−08	1E−13	94	0.02
	3	2.12E−12	3.21E−12	3.23E−12	3.21E−12	9.88−08	1E−13	109	0.02
	4	2.45E−12	3.33E−12	3.85E−12	3.33E−12	9.80E−08	1E−13	96	0.02
(4)	1	1.98E−12	5.67E−12	3.86E−12	5.67E−12	9.97E−08	1E−13	120	0.01
	2	1.79E−12	2.44E−12	2.79E−12	2.44E−12	9.94E−08	1E−13	119	0.02
	3	1.64E−12	2.33E−12	2.43E−12	2.33E−12	9.83E−08	1E−13	119	0.01
	4	1.81E−12	2.16E−12	2.58E−12	2.16E−12	9.78E−08	1E−13	116	0.01

**Table 6:** Complete numerical study of LMA-TNN for Reynolds model.

Scenario	Cases	MSE			Performance	Grad	Mu	Epochs	Time
		Training	Validation	Testing					
(1)	1	1.93E−12	2.65E−12	2.58E−12	2.65E−12	9.72E−08	1E−13	108	0.02
	2	1.20E−12	1.78E−12	1.91E−12	1.78E−12	9.96E−08	1E−13	145	0.02
	3	2.53E−12	3.64E−12	3.07E−12	3.64E−12	9.68E−08	1E−13	95	0.01
	4	2.70E−12	6.47E−12	2.91E−12	6.47E−12	9.96E−08	1E−13	111	0.01
(2)	1	1.93E−12	2.43E−12	2.37E−12	2.43E−12	9.97E−08	1E−13	119	0.02
	2	1.56E−12	2.01E−12	2.91E−12	2.01E−12	9.97E−E−08	1E−E−E−13	142	0.02
	3	3.46E−12	5.64E−12	6.10E−12	5.64E−12	9.74E−08	1E−13	105	0.02
	4	3.67E−12	5.78E−12	4.30E−12	5.78E−12	9.82E−08	1E−13	122	0.02
(3)	1	2.11E−12	3.05E−12	4.83E−12	3.05E−12	9.73E−08	1E−13	105	0.02
	2	2.07E−12	6.26E−12	3.52E−12	6.26E−12	9.86E−08	1E−13	97	0.01
	3	2.33E−13	3.50E−13	3.04E−13	3.50E−13	9.990E−08	1E−14	84	0.01
	4	1.95E−13	3.90E−13	2.74E−13	3.90E−13	9.75E−08	1E−14	92	0.01
(4)	1	1.73E−12	2.23E−12	3.68E−12	2.23E−12	9.88E−08	1E−13	121	0.02
	2	1.92E−12	3.34E−12	4.44E−12	3.34E−12	9.65E−08	1E−13	120	0.02
	3	1.87E−12	2.44E−E−E−E−12	3.84E−12	2.44E−12	9.94E−08	1E−13	114	0.02
	4	2.10E−13	2.52E−13	3.05E−13	2.52E−13	9.92E−08	1E−14	88	0.01
(5)	1	1.73E−12	2.53E−12	3.68E−12	2.53E−12	9.99E−08	1E−13	125	0.02
	2	1.92E−12	3.34E−12	4.44E−12	3.34E−12	9.64E−E−E−08	1E−14	85	0.01
	3	1.87E−12	2.44E−12	3.84E−12	2.44E−12	9.97E−08	1E−13	100	0.01
	4	2.10E−13	2.52E−13	3.05E−13	2.52E−13	9.93E−08	1E−13	97	0.01

## Conclusion

In this work, soft computing artificial intelligence is introduced using the LMA-TNN for solving the mathematical model describing the WCS-EPF with a transfer of heat and non-Newtonian Eyring–Powell fluid flow past a porous medium under the impacts of Joule heating and the magnetic field for different scenarios. Findings in brief are listed below:

- The nonlinear PDEs for the WCS-EPF are converted into nonlinear ODEs containing the heat-based changing viscosity framework.
- Runge–Kutta numerical solver is used to produce reference data for the proposed WCS-EPF in the fluid dynamics with the impacts of various physical amounts of significance such as the porous parameter, non-Newtonian parameter, magnetic parameter, thermal transfer.
- The 80 $\%$, 10 $\%$, and 10 $\%$ of the data set is chosen as validation, testing, and training for LMA-TNN. The dataset is further validated by the graphical and numerical representations in terms of convergence of the outcomes by the mean square error, the dynamics of state transition, error histograms, and regression analysis plots.
- The strong correlation between the suggested and the reference outcomes indicates the structure’s validity for all four cases of WCS-EPF, fitting of the precision $10^{-5}$ to $10^{-9}$ is also accomplished.
- We observed that the dimensionless temperature profile increases caused by the rise in the values of parameters $B_r$, *Q*, *n*, $\alpha$, and $\Omega$ while decreasing by the rise of the parameter *D*. Besides, the variation in $K_p$ does not have any observable contribution to the temperature distribution.

In the future, modern versions of artificial intelligence integrated heuristics^40–42^ will be suggested to interpret the fluid mechanics’ problems dynamics^43–46^.

## Nomenclatures

LMA: Levenberg–Marquardt algorithm
NN: Neural network
*w*: Velocity component
*Q*: Heat generation parameter
*D*, $B^*$, *O*: Viscosity parameter for Vogel’s
*m*: Viscosity parameter for Reynolds
*n*, $\alpha$: The Eyring–Powel fluid parameters
$$K_P$$: Porous parameter
$$B_r$$: Brinkman number
$\beta _n$: Non-Newtonian parameter
*c*: Constant of material
$$\theta$$: Temperature
$$\rho$$: Density
WCS: Wire coating system
A-E: Absolute error
u: Dimensionless velocity
*r*, *Z*: Space coordinates
$Q_o$: Rate of volumetric thermal generation
$$R_d$$: Die radius
Rw: Wire radius
*L*: Length
$$\theta _d$$: Saturated temperature
$$\theta _w$$: Wire temperature
$$U_w$$: Fluid velocity
$$B_o$$: Magnetic field
$$\mu$$: Shear viscosity

## References

[1] Hussain A, Ameer S, Javed F, Malik M (2019). Rheological analysis on non-Newtonian wire coating. J. Braz. Soc. Mech. Sci. Eng..

[2] Mitsoulis E (1986). Fluid flow and heat transfer in wire coating: A review. Adv. Polym. Technol..

[3] Khan NA, Sultan F, Khan NA (2015). Heat and mass transfer of thermophoretic MHD flow of Powell–Eyring fluid over a vertical stretching sheet in the presence of chemical reaction and joule heating. Int. J. Chem. Reactor Eng..

[4] Hayat T, Aslam N, Rafiq M, Alsaadi FE (2017). Hall and joule heating effects on peristaltic flow of Powell–Eyring liquid in an inclined symmetric channel. Results Phys..

[5] Ahmed A, Nadeem S (2017). Effects of magnetohydrodynamics and hybrid nanoparticles on a micropolar fluid with 6-types of stenosis. Results Phys..

[6] Khan Z (2018). Effect of magnetic field and heat source on upper-convected-Maxwell fluid in a porous channel. Open Phys..

[7] Khan Z, Rasheed HU, Ullah M, Gul T, Jan A (2019). Analytical and numerical solutions of oldroyd 8-constant fluid in double-layer optical fiber coating. J. Coat. Technol. Res..

[8] Ur Rehman F, Nadeem S (2018). Heat transfer analysis for three-dimensional stagnation-point flow of water-based nanofluid over an exponentially stretching surface. J. Heat Transf..

[9] Khan Z, Rasheed HU, Tlili I, Khan I, Abbas T (2018). Runge-kutta 4 th-order method analysis for viscoelastic oldroyd 8-constant fluid used as coating material for wire with temperature dependent viscosity. Sci. Rep..

[10] Khan Z, Khan WA, Ur Rasheed H, Khan I, Nisar KS (2019). Melting flow in wire coating of a third grade fluid over a die using Reynolds’ and Vogel’s models with non-linear thermal radiation and joule heating. Materials.

[11] Khan Z (2019). Manufacturing of double layer optical fiber coating using phan-thien-tanner fluid as coating material. Coatings.

[12] Rasheed HU (2019). Investigation of two-dimensional viscoelastic fluid with nonuniform heat generation over permeable stretching sheet with slip condition. Complexity.

[13] Khan Z (2017). Analysis of magneto-hydrodynamics flow and heat transfer of a viscoelastic fluid through porous medium in wire coating analysis. Mathematics.

[14] Khan Z (2017). Mhd flow and heat transfer analysis in the wire coating process using elastic-viscous. Coatings.

[15] Rasheed HU, Khan Z, Khan I, Ching DLC, Nisar KS (2019). Numerical and analytical investigation of an unsteady thin film nanofluid flow over an angular surface. Processes.

[16] Khan Z (2020). Heat transfer effect on viscoelastic fluid used as a coating material for wire with variable viscosity. Coatings.

[17] Sabir Z, Wahab HA, Umar M, Sakar MG, Raja MAZ (2020). Novel design of morlet wavelet neural network for solving second order lane-emden equation. Math. Comput. Simulat..

[18] Ahmad I, Raja MAZ, Bilal M, Ashraf F (2016). Bio-inspired computational heuristics to study lane-emden systems arising in astrophysics model. SpringerPlus.

[19] Raja MAZ, Ahmed T, Shah SM (2017). Intelligent computing strategy to analyze the dynamics of convective heat transfer in mhd slip flow over stretching surface involving carbon nanotubes. J. Taiwan Inst. Chem. Eng..

[20] Ahmad I (2019). Novel applications of intelligent computing paradigms for the analysis of nonlinear reactive transport model of the fluid in soft tissues and microvessels. Neural Comput. Appl..

[21] Waseem W (2020). A study of changes in temperature profile of porous fin model using cuckoo search algorithm. Alexandria Eng. J..

[22] Shah Z (2020). Design of neural network based intelligent computing for neumerical treatment of unsteady 3d flow of eyring-powell magneto-nanofluidic model. J. Mater. Res. Technol..

[23] Shoaib M (2020). Numerical investigation for rotating flow of mhd hybrid nanofluid with thermal radiation over a stretching sheet. Sci. Rep..

[24] Almalki, M. M., Alaidarous, E. S., Maturi, D., Raja, M. A. Z. & Shoaib, M. A levenberg–marquardt backpropagation neural network for the numerical treatment of squeezing flow with heat transfer model. *IEEE Access* (2020).

[25] Raja MAZ, Shah FH, Tariq M, Ahmad I (2018). Design of artificial neural network models optimized with sequential quadratic programming to study the dynamics of nonlinear troesch’s problem arising in plasma physics. Neural Comput. Appl..

[26] Raja MAZ, Manzar MA, Shah FH, Shah FH (2018). Intelligent computing for Mathieu’s systems for parameter excitation, vertically driven pendulum and dusty plasma models. Appl. Soft Comput..

[27] Masood Z, Majeed K, Samar R, Raja MAZ (2017). Design of Mexican hat wavelet neural networks for solving bratu type nonlinear systems. Neurocomputing.

[28] Mehmood A, Zameer A, Ling SH, Raja MAZ (2018). Design of neuro-computing paradigms for nonlinear nanofluidic systems of mhd jeffery-hamel flow. J. Taiwan Inst. Chem. Eng..

[29] Raja MAZ, Mehmood A, Khan AA, Zameer A (2019). Integrated intelligent computing for heat transfer and thermal radiation-based two-phase mhd nanofluid flow model. Neural Comput. Appl..

[30] Raja MAZ, Samar R, Alaidarous ES, Shivanian E (2016). Bio-inspired computing platform for reliable solution of bratu-type equations arising in the modeling of electrically conducting solids. Appl. Math. Model..

[31] Jamal R, Men B, Khan NH, Raja MAZ (2019). Hybrid bio-inspired computational heuristic paradigm for integrated load dispatch problems involving stochastic wind. Energies.

[32] Raja MAZ, Niazi SA, Butt SA (2017). An intelligent computing technique to analyze the vibrational dynamics of rotating electrical machine. Neurocomputing.

[33] Ahmad I, Raja MAZ, Bilal M, Ashraf F (2017). Neural network methods to solve the lane-emden type equations arising in thermodynamic studies of the spherical gas cloud model. Neural Comput. Appl..

[34] Raja MAZ, Umar M, Sabir Z, Khan JA, Baleanu D (2018). A new stochastic computing paradigm for the dynamics of nonlinear singular heat conduction model of the human head. Eur. Phys. J. Plus.

[35] Akbar S, Raja MAZ, Zaman F, Mehmood T, Khan MAR (2017). Design of bio-inspired heuristic techniques hybridized with sequential quadratic programming for joint parameters estimation of electromagnetic plane waves. Wirel. Pers. Commun..

[36] Faisal F, Shoaib M, Raja MAZ (2020). A new heuristic computational solver for nonlinear singular thomas-fermi system using evolutionary optimized cubic splines. Eur. Phys. J. Plus.

[37] Cheema TN (2020). Intelligent computing with levenberg-marquardt artificial neural networks for nonlinear system of covid-19 epidemic model for future generation disease control. Eur. Phys. J. Plus.

[38] Umar M (2020). A stochastic intelligent computing with neuro-evolution heuristics for nonlinear sitr system of novel covid-19 dynamics. Symmetry.

[39] Shoaib M (2021). A stochastic numerical analysis based on hybrid nar-rbfs networks nonlinear sitr model for novel covid-19 dynamics. Comput. Methods Prog. Biomed..

[40] Munir A, Manzar MA, Khan NA, Raja MAZ (2019). Intelligent computing approach to analyze the dynamics of wire coating with oldroyd 8-constant fluid. Neural Comput. Appl..

[41] Mehmood A, Afsar K, Zameer A, Awan SE, Raja MAZ (2019). Integrated intelligent computing paradigm for the dynamics of micropolar fluid flow with heat transfer in a permeable walled channel. Appl. Soft Comput..

[42] Raja MAZ, Mehmood J, Sabir Z, Nasab AK, Manzar MA (2019). Numerical solution of doubly singular nonlinear systems using neural networks-based integrated intelligent computing. Neural Comput. Appl..

[43] Imran A, Akhtar R, Zhiyu Z, Shoaib M, Zahoor Raja MA (2019). Mhd and heat transfer analyses of a fluid flow through scraped surface heat exchanger by analytical solver. AIP Adv..

[44] Umar M (2019). Numerical treatment for the three-dimensional eyring-powell fluid flow over a stretching sheet with velocity slip and activation energy. Adv. Math. Phys..

[45] Raja MAZ, Shah AA, Mehmood A, Chaudhary NI, Aslam MS (2018). Bio-inspired computational heuristics for parameter estimation of nonlinear hammerstein controlled autoregressive system. Neural Comput. Appl..

[46] Shoaib M (2019). A novel design of three-dimensional mhd flow of second-grade fluid past a porous plate. Math. Probl. Eng..

